# Use of Rapid Antigen Tests to End Isolation in a University Setting: Observational Study

**DOI:** 10.2196/45003

**Published:** 2023-04-27

**Authors:** Liliana Zigo, Alyson Wilkinson, Megan Landry, Amanda D Castel, Amita Vyas, Karen McDonnell, Nitasha Chaudhary Nagaraj, Lynn R Goldman

**Affiliations:** 1 Department of Epidemiology Milken Institute School of Public Health The George Washington University Washington, DC United States; 2 Office of the Dean Milken Institute School of Public Health The George Washington University Washington, DC United States; 3 Department of Prevention and Community Health Milken Institute School of Public Health The George Washington University Washington, DC United States; 4 Department of Environmental and Occupational Health Milken Institute School of Public Health The George Washington University Washington, DC United States

**Keywords:** COVID-19, college, university, students, rapid antigen test, isolation, antigen test, testing, tool, utility, symptoms, policy, productivity, age, university students

## Abstract

**Background:**

COVID-19 isolation recommendations have evolved over the course of the pandemic. Initially, the US Centers for Disease Control and Prevention required 10 days of isolation after a positive test result. In December 2021, this was reduced to a minimum of 5 days with symptom improvement, followed by 5 days of mask wearing. As a result, several institutions of higher education, including the George Washington University, required persons testing positive for COVID-19 to either submit a negative rapid antigen test (RAT) with symptom resolution to leave isolation after 5 days or to maintain a 10-day isolation period in the absence of a negative RAT and the presence of continued symptoms. RATs are tools that can be used both to shorten isolation periods and to ensure that persons testing positive for COVID-19 remain in isolation if infectious.

**Objective:**

The purpose of this analysis is to report on the experience of implementing RAT policies, examine the number of days that isolation was reduced via RAT testing, determine the factors that predicted uploading a RAT, and determine RAT positivity percentages to illustrate the utility of using RATs to end isolation.

**Methods:**

In this study, 880 individuals in COVID-19 isolation at a university in Washington, DC, uploaded 887 RATs between February 21 and April 14, 2022. Daily positivity percentages were calculated, and multiple logistic regression analyses examined the odds of uploading a RAT by campus residential living status (ie, on or off campus), student or employee designation, age, and days in isolation.

**Results:**

A total of 76% (669/880) of individuals in isolation uploaded a RAT during the study period. Overall, 38.6% (342/887) of uploaded RATs were positive. Uploaded RATs were positive 45.6% (118/259) of the time on day 5; 45.4% (55/121) on day 6; 47.1% (99/210) on day 7; and 11.1% (7/63) on day 10 or beyond. Adjusted logistic regression modeling indicated cases living on campus had increased odds of uploading a RAT (odds ratio [OR] 2.54, 95% CI 1.64-3.92), whereas primary student affiliation (OR 0.29, 95% CI 0.12-0.69) and days in isolation (OR 0.45, 95% CI 0.39-0.52) had decreased odds of uploading a RAT. Of the 545 cases with a negative RAT, 477 were cleared prior to day 10 of their isolation due to lack of symptoms and timely submission, resulting in a total of 1547 days of lost productivity saved compared to all being in isolation for 10 days.

**Conclusions:**

RATs are beneficial, as they can support a decision to release individuals from isolation when they have recovered and maintain isolation for people who may still be infectious. Future isolation policies should be guided by similar protocols and research to reduce the spread of COVID-19 and minimize lost productivity and disruption to individuals’ lives.

## Introduction

More than 2 years into the COVID-19 pandemic, research and policy recommendations continue to evolve. Initially, the US Centers for Disease Control and Prevention (CDC) required 10 days of isolation after a positive test result, contingent upon being fever-free for 72 hours with symptoms improving [1]. In December 2021, the CDC reduced the required isolation period to a minimum of 5 days as long as symptoms were resolving, followed by 5 days of wearing a close-fitted mask in public spaces and around others [2]. A negative COVID-19 test was not required to end isolation at 5 days [2]. However, concerns mounted that this could result in individuals leaving isolation prematurely, as an estimated one-third of individuals may continue to be infectious [3].

Rapid antigen tests (RATs) are used to detect a COVID-19 infection, having the advantage of providing quick results and the ability to be used at home [4]. However, RATs have a lower sensitivity than a polymerase chain reaction (PCR) test, requiring a higher viral load for a positive test result [5]. Previous studies have demonstrated RAT positivity rates of 31%-58% after at least 5 days of isolation [3], suggesting a strategy based on a negative RAT and symptom resolution to reduce the number of individuals leaving isolation while still infectious as well as reducing the number of people who remain unnecessarily in isolation [6,7]. A study of a health care worker’s return-to-work antigen testing program found that antigen test positivity was 60.5% at day 5 and 47.4% positive at day 7 [8]. A second study of college athletes found that after 7 days of isolation, 27% continued to test positive on rapid antigen tests; it concluded that a 5-day isolation period may be insufficient [9].

As a result, several institutions of higher education [10,11], including the George Washington University (GW), required persons testing positive for COVID-19 to either submit a negative RAT with symptom resolution to leave isolation after 5 days or to maintain a 10-day isolation period in the absence of a negative RAT and the presence of continued symptoms.

Due to rising infection rates during the first Omicron wave, GW started the first 2 weeks of the spring (January 2022) semester virtually and required that university members obtain a PCR test or RAT when arriving on campus. GW mandated that all those on campus be tested twice monthly for those who were fully vaccinated and weekly for those with a vaccine or booster exemption. The GW Public Health Laboratory provided COVID-19 PCR testing on campus; however, university community members could report external PCR or RAT positive results to the internal GW medical portal.

On January 28, 2022, GW modified the required isolation time from 10 to 7 days per guidance from DC Health [12]. Effective February 22, 2022, GW began accepting a negative RAT for the purpose of ending isolation at 5 days and returned to a 10-day isolation period in the absence of a negative RAT or continued symptoms. Day 1 of isolation was defined as the test collection date or the symptom onset date, whichever was earlier. Antigen tests were provided for those who were isolating, and antigen tests approved by the US Food and Drug Administration [13] were accepted by GW for the purpose of isolation clearance. In February, GW recommended testing at day 5 and then again at day 7 if the first RAT was positive. On April 14, 2022, GW revised the RAT protocol to accept a negative test any day after day 5 of isolation with symptom improvement. Some cases submitted a RAT following day 10 of isolation due to lingering symptoms keeping them in isolation. All persons who tested positive for SARS-CoV-2 were required to complete a daily isolation survey, and those who completed a RAT uploaded their results to the GW medical portal. A GW medical provider reviewed daily surveys and RAT results to determine isolation clearance.

The purpose of this analysis is to report on the experience of implementing these policies and examine the number of days that isolation was reduced via RAT testing; this analysis also aims to examine the factors that predicted uploading a RAT and determine RAT positivity percentages to illustrate the utility of using RATs to end isolation.

## Methods

### Participants and Data Collection

The GW COVID-19 case management system embedded in the Student Health medical record system was used to evaluate the data we collected relevant to RAT testing for people who were in isolation. There were 955 cases identified between February 21 and April 14, 2022. Cases with the following attributes were removed from the analysis: a positive test uploaded more than 7 days into the isolation period (n=34), false positives (n=2), no initial positive listed in the patient chart (n=2), individuals lost to follow-up (n=8), individuals who uploaded a PCR for purposes of isolation clearance (n=3), those with a positive test within 90 days of a previous positive test (n=4), and those who were medically released before 10 days based on verbal confirmation of negative antigen tests (n=22). The final analytic sample was 887 RAT results among 880 cases.

### Statistical Analysis

Descriptive statistics were computed for all variables. Categorical variables, including campus residential living status and student or employee designation, were described using frequencies and percentages. Continuous variables, including age and number of days in isolation, were described using medians and IQRs. Next, we calculated positive percentages for the day a RAT was submitted to identify if individuals were eligible to be released before the end of the 10-day isolation period. Then, simple logistic regressions were run to compute the odds of uploading a RAT by individual independent variables, including campus residential living (ie, on or off campus), student or employee designation, age, and days in isolation. A final multivariable logistic regression model computed the odds of uploading a RAT with the independent variables simultaneously, including campus residential living status (on or off campus), student or employee designation, age, and days in isolation. SAS (version 9.4) was used for all analyses.

### Ethical Considerations

All community members provided informed consent to participate in the GW COVID-19 surveillance program, and the GW Institutional Review Board concluded that these were nonresearch-related activities.

## Results

The sample included 43.6% (384/880) of persons living on campus and 56.4% (496/880) of persons living off campus. Students accounted for 87.8% (773/880) of the sample, and 12.2% (107/880) were employees. The median age was 21.8 (IQR 20.3-25.7) years, and the mean number of days in isolation was 8.5 days. Table 1 presents the population demographics of the students and employees with COVID-19 in our final analytical sample.

**Table 1:** Population demographics (N=880).

Characteristics			Total (N=880)		Antigen test (n=669)		No antigen test (n=211)
**Living status, n (%)**							
	Off campus	496 (56.4)		340 (50.8)		156 (73.9)	
	On campus	384 (43.6)		329 (49.2)		55 (26.1)	
**Affiliation, n (%)**							
	Employee	107 (12.2)		84 (12.6)		23 (10.9)	
	Student	773 (87.8)		585 (87.4)		188 (89.1)	
Age (years), median (IQR)			21.8 (20.3-25.7)		21.5 (20-25.2)		22.6 (21.2-27)
Days in isolation, median (IQR)			9 (7-10)		8 (6-10)		10 (10-11)

A total of 76% (669/880) of individuals uploaded a RAT during the study period with the majority (476/880, 54.1%) uploading 1 test, 19.2% (169/880) uploading 2 tests, and 2.7% (24/880) uploading 3 or more tests. Overall, 39% (342/887) of all RATs were positive. Table 2 presents the percent positive by test number and day of isolation. Positivity percentages for RATs were 45.6% (118/259) on day 5; 45.4% (55/121) on day 6; 47.1% (99/210) on day 7; and 11.1% (7/63) on day 10 or beyond. Second tests, aggregated across all days, had 45.1% (87/193) that remained positive; 12.5% (3/24) of third tests, aggregated across all days, remained positive.

**Table 2:** Percent positive by test number and day of isolation at the George Washington university in Washington, DC.

Day of isolation	In isolation, n (%)	Tests per day	Negative tests per day	Person-days saved from isolation	Daily test (positive), n (%)	Test 1 (positive), n/N (%)	Test 2 (positive), n/N (%)	Test 3 (positive), n/N (%)	Test 4 (positive), n/N (%)
0	880 (100)	0	—^a^	—	—	—	—	—	—
3^b^	880 (100)	4	4	—	0 (0)	0/4 (0)	—	—	—
4^b^	880 (100)	12	8	—	4 (33.3)	4/11 (36.4)	0/1 (0)	—	—
5	739 (84)	259	141	705	118 (45.6)	118/255 (46.3)	0/4 (0)	—	—
6	673 (76.5)	121	66	264	55 (45.5)	52/110 (47.3)	3/11 (27.3)	—	—
7	562 (63.9)	210	111	333	99 (47.1)	43/113 (38.1)	55/95 (57.9)	1/1 (100)	0/1 (0)
8	476 (54.1)	127	86	172	41 (32.3)	21/79 (26.6)	20/42 (47.6)	0/6 (0)	—
9	403 (45.8)	91	73	73	18 (19.8)	8/56 (14.3)	9/24 (37.5)	1/11 (9.1)	—
≥10^c^	347 (39.4)	63	56	—	7 (11.1)	6/41 (14.6)	0/16 (0)	1/6 (16.7)	—
Total	—	887	545	1547	342 (38.6)	252/669 (37.7)	87/193 (45.1)	3/24 (12.5)	0/1 (0)

^a^Not applicable.

^b^Contrary to policy and advice, some submitted rapid antigen test tests prior to the fifth day of isolation.

^c^Contrary to policy, some continued rapid antigen test testing on day 10 of isolation and beyond.

Assuming all cases were cleared from isolation by a medical provider the same day they uploaded a negative antigen test, those who uploaded a negative result on days 5-9, a total of 477 cases were cleared and were not required to isolate for the full 10 days. This resulted in 1547 days or approximately 4.2 years of productivity, classes, or days “saved” and not in isolation. For example, a case who uploaded a negative RAT on day 6 and was subsequently cleared would result in 4 days “saved” that, without the test-out protocol, would have remained in isolation [14]. By day 7 and by day 10 and beyond (Table 2), an estimated 63.9% (562/880) and 39.4% (347/880) continued to be in isolation because of continued test positivity, symptoms, or both. On those days, RAT test positivity (among those testing) was 47.1% (99/210) and 11.1% (7/63), respectively.

Table 3 presents characteristics associated with the odds of uploading a RAT over the course of the study. Unadjusted logistic regression found that on-campus residents had increased odds of uploading a RAT, and those with more days in isolation had lower odds of uploading a RAT. The final adjusted logistic regression model provided similar estimates. Cases living on campus had increased odds of uploading a RAT (odds ratio [OR] 2.54, 95% CI 1.64-3.92); students (compared to employees) had lower odds of uploading a RAT (OR 0.29, 95% CI 0.12-0.69); and those with more days in isolation had lower odds of uploading a RAT (OR 0.45, 95% CI 0.39-0.52).

**Table 3:** Characteristics associated with the odds of uploading a RAT over the course of the study among university members using an adjusted multivariable logistic regression model (N=880).

Characteristics		Unadjusted OR^a^ (95% CI)	Adjusted^b^ OR (95% CI)
**Living status**			
	Off campus	Reference	Reference
	On campus	2.75 (1.95-3.87)	2.54 (1.64-3.92)
**Primary affiliation**			
	Employee	Reference	Reference
	Student	0.85 (0.52-1.39)	0.29 (0.12-0.69)
Age (years)		0.98 (0.97-1.00)	1.00 (0.97-1.04)
Days in isolation (1 day)		0.46 (0.41-0.53)	0.45 (0.39-0.52)

^a^OR: odds ratio.

^b^Adjusted for all other variables listed in the column.

## Discussion

### Principal Findings

This analysis sought to evaluate the utility of using RATs to end isolation periods more safely prior to the full 10 days of isolation. Rapid antigen tests are beneficial, as they provide valuable insight to guide isolation and treatment, allowing health care providers to release individuals from isolation when they are no longer contagious and maintain isolation for people who may still be infectious. In this analysis, the percentage of positive RATs uploaded on day 5 of isolation was 45.6% (118/259). If isolation were set to only 5 days, most likely a proportion of the 45.6% who uploaded a positive RAT would still be infectious and able to spread COVID-19 on campus and in the community despite advice to employ one-way masking [15]. This result is consistent with prior studies conducted in various settings ranging from Mayo Clinic Florida’s COVID Virtual Clinic to other universities in the United States, which have reported RAT positivity rates of 26%-68% at day 5 [1,6,10]. Of note, the percentage of RAT uploads that were positive at day 7 (99/210, 47.1%) is no lower than those at day 5, as opposed to day 10 and beyond, when only 11.1% (7/63) were positive. Even in the context of fairly high positive percentages, the RAT testing strategy resulted in 477 cases being cleared prior to day 10 of their isolation or 1547 days of lost productivity saved.

After adjusting for confounders, living status, days in isolation, and primary affiliation were all significantly associated with uploading a RAT. All those living on campus were students, and they may have had higher odds of uploading a RAT because they were provided RATs in person. Off-campus cases had the option to either pick RATs up from an on-campus location or have a testing kit mailed to them, possibly resulting in delays, which may have made it more challenging for certain off-campus cases to complete a RAT when recommended. Employees were more likely to upload RATs, possibly due to reasons such as wanting to return to work or running out of pandemic leave or sick time. Additionally, occupational health followed up with cases through phone calls multiple times during their isolation, whereas the student health center was unable to do so. Certainly, a testing out of isolation strategy should ensure that all members of the population have equitable access to RATs as well as follow-up medical supervision.

### Strengths

Although this is not the first study to analyze a COVID-19 RAT protocol, this study has two unique strengths. First, the sample size was significantly larger than several similar studies, which ranged from 40 to 323 [6,10,16,17]. The CDC published a study analyzing 3502 COVID-19 infections, with 729 antigen tests in their study [18]. Second, previous studies among institutions of higher education have focused solely on students [10], while this study was able to assess the experience with both students and employees. Additionally, the use of RATs allows for individuals to return to work or class sooner than they would otherwise be allowed to return. This benefit is particularly important for a university setting because missing a class can be detrimental to academic achievement and can increase stress during an already potentially stressful time [19].

### Limitations

There are several limitations to this study. First, this study relied on cases self-reporting the results of their RATs, and we do not know why 24% (211/880) of our cases did not do so (whether related to lack of access to the tests, opting not to do the tests, failure to follow instructions to upload the RAT test results, or having symptoms throughout, such that the test results would have been irrelevant). Almost certainly, more cases took a RAT than GW was aware of, which could skew the results, depending on why the RATs were not uploaded. For those with asymptomatic infections, either at the outset or over the entire course of the illness, it is difficult to ascertain when they acquired their infection [18]. Thus, asymptomatic cases may be more likely to have a negative RAT as additional days may have elapsed since the onset of their infection. Compared to prior studies [6,10,16,18], this study did not perform analyses based on vaccine status, as only 2 cases had vaccine exemptions.

### Conclusions

The most important issue is how to best isolate people with COVID-19 to balance public health protection with sparing individuals’ needless days in isolation. This study, and many others, support the notion that automatically ending isolation at 5 days is risky in terms of sending many who are infectious back into the community, in fact, that 7 days may be too short as well. Yet many can come out of isolation earlier with RAT testing. Many questions remain, including the efficacy of one-way masking for those coming out of isolation who still are RAT positive (as occurs with the current CDC protocol) and the need to document transmission of COVID-19 from people who have been isolated fewer than 10 days. Additionally, for both asymptomatic and symptomatic cases, RATs may not be the best measure of infectiousness [6,15,20,21], possibly resulting in the premature release of cases from isolation or an unnecessary extension of isolation. These research avenues, along with findings from this study, can guide the development of better-informed isolation policies that are protective of public health and cause the least disruption to individuals’ lives.

## Abbreviations

CDC: Centers for Disease Control and Prevention
GW: George Washington University
OR: odds ratio
PCR: polymerase chain reaction
RAT: rapid antigen test

## Data Availability

The data sets generated or analyzed during this study are available from the corresponding author on reasonable request.
